# Supplementary material for: Astragalus polysaccharide (PG2) Ameliorates Cancer Symptom Clusters, as well as Improves Quality of Life in Patients with Metastatic Disease, through Modulation of the Inflammatory Cascade
Source: Cancers (Basel). 2019 Jul 25;11(8):1054. doi: 10.3390/cancers11081054 (PMC6721312; doi:10.3390/cancers11081054)
Supplement: Supplementary file 1 [file cancers-11-01054-s001.pdf]

Supplementary Materials

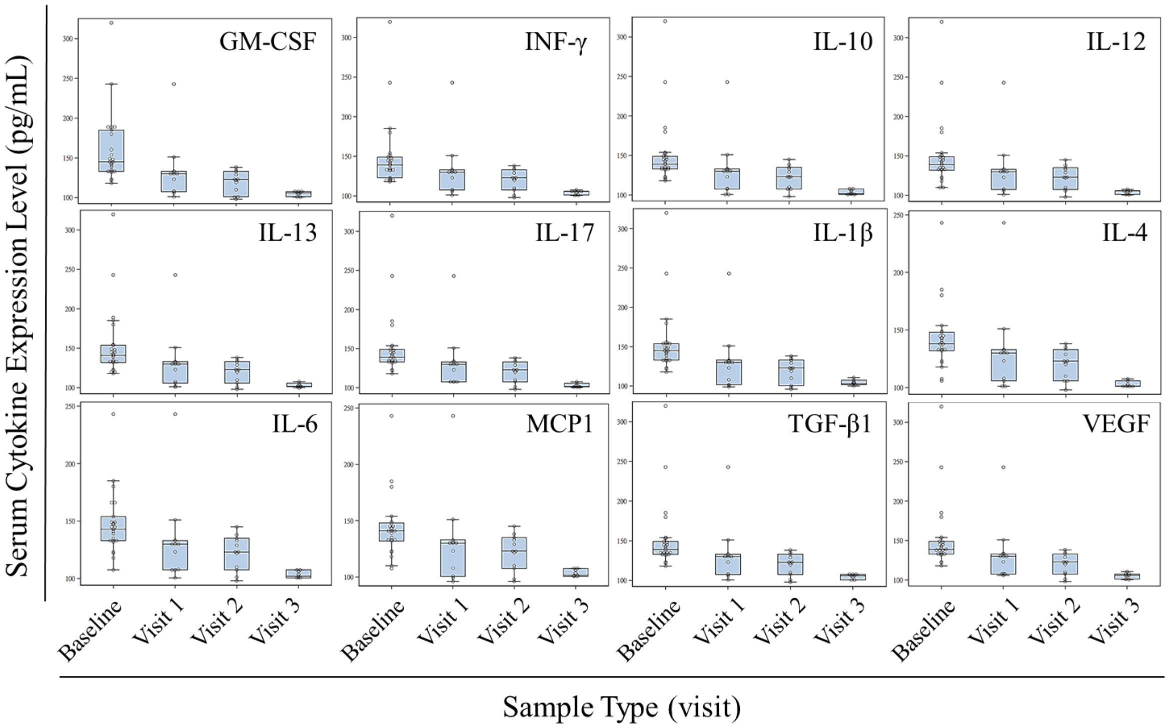

Figure S1. Graphical representation of the individual inflammatory cytokine level in each patient from our advanced progressive metastatic cancer cohort.

Table S1. PG2-induced alteration in patients' inflammatory cytokine profile at baseline, first, and last visits.

		Baseline	v1	v3
IL-1β	High	154.8 ± 33	130.4 ± 15.27	103.13 ± 3.19
	Low	156.23 ± 54.25	132.97 ± 55.44	106.1 ± 4.55
	combined	155.5 ± 44.3	131.8 ± 40.4	104.4 ± 3.8
IL-4	High	153.35 ± 33.39	130.4 ± 15.27	101.31 ± 0.24
	Low	133.61 ± 20.44	134.07 ± 54.7	104.86 ± 3.29
	combined	143.1 ± 28.6	132.4 ± 39.9	102.8 ± 2.7
IL-6	High	155.9 ± 33.17	130.4 ± 15.27	102.82 ± 3.19
	Low	139.54 ± 20.6	135.3 ± 53.9	104.66 ± 3.64
	combined	147.4 ± 28	133.1 ± 39.4	103.6 ± 3.2
MCP1	High	153.63 ± 33.26	130.4 ± 15.27	104.2 ± 3.85
	Low	134.21 ± 20.03	131.95 ± 56.17	102.7 ± 2.8
	combined	143.5 ± 28.3	131.2 ± 40.9	103.6 ± 3.3
IL-10	High	153.08 ± 33.54	130.4 ± 15.27	104.2 ± 3.85
	Low	152.25 ± 55.5	134.14 ± 54.68	102.7 ± 2.8
	combined	152.6 ± 45.3	132.4 ± 39.9	103.6 ± 3.3
IL-12	High	150.81 ± 35.66	130.4 ± 15.27	104.96 ± 3.03
	Low	151.41 ± 56.1	135.12 ± 53.99	103.02 ± 2.5
	combined	151.1 ± 46.4	133 ± 39.5	104.1 ± 2.8
IL-13	High	151.72 ± 34.63	130.4 ± 15.27	102.48 ± 3.41
	Low	156.75 ± 56.16	134.15 ± 54.64	103.18 ± 2.34
	combined	154.3 ± 46.1	132.4 ± 39.9	102.8 ± 2.8
IL-17	High	153.08 ± 33.54	130.4 ± 15.27	101.16 ± 0.45
	Low	153.75 ± 54.79	136.46 ± 53.07	105.13 ± 2.84
	combined	153.4 ± 44.9	133.7 ± 38.9	102.9 ± 2.7
GM-CSF	High	157.99 ± 34.56	130.4 ± 15.27	104.2 ± 3.85
	Low	162.5 ± 55.59	135.12 ± 53.99	106.42 ± 0.96
	combined	160.3 ± 45.8	133 ± 39.5	105.2 ± 3
VEGF	High	153.35 ± 33.39	130.4 ± 15.27	103.79 ± 3.44
	Low	154 ± 54.7	136.18 ± 53.25	108.06 ± 2.49
	combined	153.7 ± 44.8	133.6 ± 39	105.6 ± 3.6
TGF-β1	High	153.08 ± 33.54	130.4 ± 15.27	105.79 ± 3.48
	Low	153.5 ± 54.88	135.02 ± 54.07	104.1 ± 3.06
	combined	153.3 ± 44.9	132.9 ± 39.5	105.1 ± 3.2
IFN-γ	High	151.72 ± 34.63	130.4 ± 15.27	104.96 ± 3.03
	Low	151 ± 56.09	135.12 ± 53.99	104.31 ± 2.7
	combined	151.3 ± 46	133 ± 39.5	104.7 ± 2.7
